# Evaluating the Use of Vincristine as a Substitute in Hodgkin Lymphoma Treatment

**DOI:** 10.7759/cureus.84276

**Published:** 2025-05-17

**Authors:** Mousa Atmeh, Mohammad S Al-Saudi, Tareq M El-Ali, Aasem Rawshdeh, Anfal N Daheerat, Abed AlFattah H AlNsour, Zaid Sarhan, Ahmad Alsharawneh, Osama Alghafri, Husam M Alzghoul, Ala' W Matalqa, Dia Sarhan, Raneem Dalaeen, Abdel-Rahman Al Husni, Mosab Atmeh

**Affiliations:** 1 Department of Clinical Oncology, Jordanian Royal Medical Services, Amman, JOR; 2 Department of Neurology, Jordanian Royal Medical Services, Amman, JOR; 3 Department of Radiation Oncology, Jordanian Royal Medical Services, Amman, JOR; 4 Department of Internal Medicine, Jordanian Royal Medical Services, Amman, JOR; 5 General Medicine, Jordan University Hospital, Amman, JOR; 6 Internal Medicine, Jordan Medical University, Amman, JOR; 7 Oncology, Jordan University for Science and Technology, Amman, JOR

**Keywords:** abod regimen, hodgkin lymphoma, relapse risk, treatment response, vincristine

## Abstract

Introduction

Classical Hodgkin lymphoma (HL) is a type of cancer originating from B lymphocytes, characterized by a high annual incidence globally. While the ABVD regimen (adriamycin, bleomycin, vinblastine, and dacarbazine) is the standard treatment, this study aims to evaluate the efficacy of the ABOD regimen (adriamycin, bleomycin, vincristine, and dacarbazine) in treating classical HL. The study also aimed to identify factors influencing treatment response, relapse risk, and the indication for alternative treatments such as escalation therapy, salvage therapy, immunotherapy, and transplantation.

Methods

This ambispective study included 81 patients with classical HL treated with the ABOD regimen containing vincristine at the Military Cancer Center (MCAC) in Amman, Jordan, between January 2017 and January 2022, with follow-up until February 2023. Patients aged 14 to 71 years, with disease stages 2a to 4b and performance status scores of 0 to 2, were included. Exclusion criteria were age below 14, loss to follow-up, stage 1a disease, or performance score >2. Data were primarily collected from electronic records and clinical notes. Treatment response after the second and sixth chemotherapy cycles, and the need for escalation, salvage therapy, or bone marrow transplantation were assessed. Statistical analysis included Chi-square tests for categorical variables and ANOVA and t-tests for continuous variables, followed by logistic regression for significant predictors. Ethical approval was obtained from the IRB of the Jordanian Royal Medical Services.

Results

The study included 81 patients (n = 81), with a mean age of 34.79 years and a predominance of females (n = 49, 60.5%) compared to males (n = 32, 39.5%). The most common histological subtype was nodular sclerosis (n = 67, 82.7%), and the most prevalent stage was stage 4 (n = 31, 38.3%). Treatment response after the second cycle of ABOD was significantly better in older patients. There was no significant association between bulky disease (n = 20, 24.7%) and treatment response or relapse risk (n = 27, 33.3%). Younger patients required dose escalation more frequently (n = 24, 29.6%), suggesting that the ABOD regimen might be more suitable for older patients. Gender, histological subtype, and disease stage were not significantly associated with treatment response or relapse risk.

Conclusion

The ABOD regimen, using vincristine instead of vinblastine, presents a viable alternative to the ABVD regimen for classical HL treatment, particularly benefiting older patients due to favorable response rates and manageable toxicity. The findings highlight the need for further research to optimize treatment strategies for different patient subgroups and confirm the benefits of the ABOD regimen.

## Introduction

Hodgkin lymphoma (HL) is a B-cell neoplasm of the immune system [1]. The incidence of HL is approximately 80,000 cases annually worldwide and is expected to increase to more than 107,000 cases by 2040 [2]. Advances in treatment modalities for HL have raised the 10-year survival rate to over 80% [3].

According to the World Health Organization (WHO) 2008 classification, there are two histological types of HL: nodular lymphocyte predominant and classical HL. Classical HL has four subtypes: nodular sclerosis, mixed cellularity, lymphocyte depletion, and lymphocyte-rich lymphoma [4]. Various factors, such as small family size, single-family housing, and high maternal education, have been associated with HL in the young population [5]. In addition, Epstein-Barr virus infection significantly increases the risk of developing HL [6].

The standard chemotherapy regimen for HL is adriamycin, bleomycin, vinblastine, and dacarbazine (ABVD), which is associated with reduced toxicity compared to older regimens such as MOPP regimen (mechlorethamine, vincristine, procarbazine, and prednisone) [7,8]. Vincristine, which is also a component of the BEACOPP regimen (bleomycin, etoposide, doxorubicin, cyclophosphamide, vincristine, procarbazine, and prednisone), effectively induces microtubule depolymerization and mitotic arrest. BEACOPP demonstrates superior efficacy compared to ABVD but is associated with higher toxicity [9,10].

In this study, vincristine was used as part of the ABOD regimen (doxorubicin, bleomycin, dacarbazine, and vincristine) instead of vinblastine. We aimed to identify factors such as age, sex, histological subtype, and stage and their relationship with treatment response to vincristine and the risk of relapse. We also studied factors associated with the need for escalation, salvage therapy, transplantation, and immunotherapy.

## Materials and methods

This study used an ambispective design to evaluate the effectiveness of substituting vinblastine with vincristine in the ABOD regimen (adriamycin, bleomycin, vincristine, and dacarbazine) for the treatment of classical HL. The study involved analyzing data retrospectively and prospectively from patients treated between January 2017 and January 2022 at the Military Cancer Center (MCAC), a specialized oncology unit under the Jordanian Royal Medical Services, located in Amman, Jordan, with follow-up conducted until February 2023.

All patients included in this study were treated using the ABOD regimen containing vincristine. The study included patients diagnosed with classical HL, aged between 14 to 71 years, with a performance status score ranging from 0 to 2, and disease stages from 2a to 4b. Patients younger than 14 years, those who were lost to follow-up, patients with poor performance status (score > 2), or those diagnosed with stage 1a classical HL were excluded from the analysis.

The data were primarily collected from the hospital’s electronic medical records and clinical notes. Secondary data sources included physical documentation and reports provided by patients. Data completeness and accuracy were carefully reviewed and verified.

The effectiveness of the ABOD regimen was assessed by evaluating the treatment response at two key points: an initial response after the second chemotherapy cycle and a long-term response after the sixth cycle. The primary outcome measure was the achievement of a complete treatment response, determined using the Deauville score based on PET scan findings. Patients with a Deauville score of 3 or lower after cycle 2 were considered to have responded to chemotherapy and were managed by de-escalating treatment through the removal of bleomycin. Conversely, patients with a Deauville score of 4-5 underwent biopsy to confirm active disease and, if positive, were escalated to the BEACOPP regimen. At the end of cycle 6, the treatment response was again assessed using PET scan and, if needed, biopsy. Patients with confirmed relapsed or refractory disease at this stage were considered for salvage therapy and, if eligible, autologous bone marrow transplantation. Non-eligible patients proceeded to immunotherapy as a subsequent line of treatment.

To explore associations with the need for treatment escalation, salvage therapy, transplantation, and immunotherapy, the following factors were considered: Deauville score from PET scan, performance status, patient age, frailty status, presence of comorbidities, and bone marrow transplant (BMT) risk score.

IBM SPSS Statistics for Windows, Version 21.0 (released 2012, IBM Corp., Armonk, NY) was employed for statistical analysis. Initially, categorical variables including sex, histological subtype, disease stage, treatment escalation, and salvage therapy were analyzed using Chi-square tests. Continuous variables, particularly age, were evaluated for differences among groups using ANOVA or independent-samples t-tests as appropriate. Variables demonstrating statistical significance in these preliminary analyses were then included in logistic regression models to further explore associations with treatment response. Statistical significance was determined at a p-value threshold of <0.05.

This study was conducted following routine clinical practice guidelines and ethical standards. This study was approved by the Royal Medical Services Human Research Ethics Committee under protocol number 6/2024, in a meeting held on May 7, 2024. The approval confirms compliance with ethical standards, and consent for treatment and open access publication was obtained or waived for all participants.

## Results

Sociodemographic and clinical characteristics

The study enrolled a total of 81 patients, with a slight predominance of females (n = 49, 60.5%) over males (n = 32, 39.5%). The mean age of the participants was 34.79 years, with a standard deviation of 14.82. Regarding disease stage, the majority of patients were diagnosed at advanced stages, with stage 4b being the most frequent (n = 31, 38.3%), followed by stage 2b (n = 15, 18.5%) and stage 2a (n = 12, 14.8%). Histopathologically, nodular sclerosis subtype was dominant (n = 67, 82.7%), while mixed cellularity and lymphocyte depletion were reported in 13.6% (n = 11) and 3.7% (n = 3) of patients, respectively. Bulky disease was present in 24.7% of the cohort (n = 20). In addition, 33.3% (n = 27) of the patients required salvage therapy, and 29.6% (n = 24) needed treatment escalation. The relapse rate was also 33.3% (n = 27). As for the treatment response, 66.7% (n = 54) of the patients achieved complete response after the second cycle, and this increased to 80.2% (n = 65) after completing six cycles of chemotherapy. These clinical characteristics are summarized in Table 1.

**Table 1:** Sociodemographic and clinical characteristics of the participants.

Variable	Category	Frequency	Percentage
Sex	Females	49	60.5%
	Males	32	39.5%
Stage	Stage 2a	12	14.8%
	Stage 2b	15	18.5%
	Stage 3a	8	9.9%
	Stage 3b	9	11.1%
	Stage 4a	6	7.4%
	Stage 4b	31	38.3%
Subtype	Nodular sclerosis	67	82.7%
	Mixed cellularity	11	13.6%
	Lymphocyte depletion	3	3.7%
Bulky disease	No	61	75.3%
	Yes	20	24.7%
Need for salvage therapy	No	54	66.7%
	Yes	27	33.3%
Need for escalation	No	57	70.4%
	Yes	24	29.6%
Relapse	No	54	66.7%
	Yes	27	33.3%
Post-cycle two complete responses	No	27	33.3%
	Yes	54	66.7%
Post-cycle six complete responses	No	16	19.8%
	Yes	65	80.2%
Age range	10–19 years	8	10.0%
	20–29 years	30	37.5%
	30–39 years	18	22.5%
	40–49 years	10	12.5%
	50–59 years	10	12.5%
	Mean (SD)	34.79	(14.82)

Response analysis by age and clinical variables

Table 2 presents the statistical comparisons of age across key clinical groups. Patients who achieved a complete response after two cycles were significantly older (mean age 37.78 ± 15.44 years) than those who did not respond (mean age 28.81 ± 11.60 years), with a p-value of 0.009. Similarly, patients who required therapy escalation were younger (28.42 ± 11.30 years) than those who did not (37.47 ± 15.38 years), with a significant p-value of 0.011. A statistically significant difference was also observed in age between males and females (p = 0.048) and between different lymphoma subtypes (p = 0.039). These findings are summarized in Table 2.

**Table 2:** Asssociations between patient characteristics and response to treatment.

Variables	Category	N	Mean age	SD	Test value	P-value
Post-cycle two complete responses	No	27	28.81	11.60	2.662	0.009
	Yes	54	37.78	15.44		
Needs of escalation	No	57	37.47	15.38	2.601	0.011
	Yes	24	28.42	11.30		
Gender	Females	49	32.16	13.46	2.012	0.048
	Males	32	38.81	16.07		
Subtypes	Nodular sclerosis (a)	67	33.45	13.53	3.377	0.039
	Mixed cellularity (b)	11	44.91	19.92		
	Lymphocyte depletion	3	27.67	7.51		
	LSD post-hoc test		B vs A	–	–	0.017

Association between salvage therapy and treatment response

As shown in Table 3, a significant association was observed between the need for salvage therapy and complete response after six cycles. Among patients who required salvage therapy (n = 32), those who failed to achieve complete response were the majority (n = 30, 93.8%), compared to only 18.5% (n = 10) among those who did not require salvage therapy (n = 54). This association was statistically significant (χ² = 32.750, p < 0.001).

**Table 3:** Association between post-cycle six complete response and the need for salvage therapy.

Variables	Category	No (n, %)	Yes (n, %)	Chi-square	P-value
Needs of salvage	No	10 (18.5%)	44 (81.5%)		
	Yes	15 (93.8%)	1 (6.3%)	32.750	<0.001

Relationship between lymphoma subtype and disease stage

The distribution of HL subtypes across disease stages revealed that nodular sclerosis was predominant in all stages, particularly in stage 4b (n = 29, 43.3%). Mixed cellularity subtype was relatively equally distributed across early and advanced stages but constituted a small fraction overall (n = 11, 13.6%). Lymphocyte depletion subtype was rare and only observed in stage 4a (n = 1, 1.2%) and stage 4b (n = 2, 2.5%). The observed distribution was statistically significant, with a p-value of 0.042 using the Fisher-Freeman-Halton exact test (Table 4). The test used was Fisher-Freeman-Halton exact test (χ² = 14.558, df = 10, p = 0.042).

**Table 4:** Distribution of lymphoma subtypes across disease stages.

Stage	Nodular sclerosis n (%)	Mixed cellularity n (%)	Lymphocyte depletion n (%)
Stage 2a	9 (13.4%)	3 (27.3%)	0 (0.0%)
Stage 2b	12 (17.9%)	3 (27.3%)	0 (0.0%)
Stage 3a	6 (9.0%)	2 (18.2%)	0 (0.0%)
Stage 3b	8 (11.9%)	1 (9.1%)	0 (0.0%)
Stage 4a	3 (4.5%)	1 (9.1%)	2 (66.7%)
Stage 4b	29 (43.3%)	1 (9.1%)	1 (33.3%)

## Discussion

This section reviews the efficacy and clinical outcomes of the ABOD regimen in HL, particularly its impact on response rates, escalation, and relapse. To our knowledge, this study is among the few that evaluate vincristine-based ABOD therapy (vincristine, doxorubicin, bleomycin, and dacarbazine) in this context. Bulky disease, defined as a single nodal mass ≥10 cm or mediastinal involvement exceeding one-third of the transthoracic diameter [11], is generally considered an unfavorable prognostic factor [12,13]. However, our findings showed no significant association between bulky disease and age, sex, treatment response, or relapse risk, in contrast to previous studies linking it to younger age and poor response under ABVD treatment [11,14]. These findings highlight the need for further research to better characterize the prognostic implications of bulky disease in patients receiving alternative regimens like ABOD.

Regarding the effect of sex on treatment response and the risk of relapse, no significant association was found between gender, treatment response to ABOD regimen after the sixth cycle, and relapse in our study. Other studies found that gender was not significantly associated with treatment response to the ABVD regimen, even though low rates of relapse have been observed among female patients compared to male patients [15]. Male sex is a poor prognostic factor in patients with HL [16]; thus, more studies are needed to understand the physiological mechanism behind being male and its association with poor prognosis. Our study showed that age was significantly associated with better treatment response after the second cycle of the ABOD regimen, with older patients showing a higher response rate. Other studies have shown no difference in treatment response to ABVD regimens with age [17]. Although age is a poor prognostic factor in HL patients, this could be explained by the overall increase in non-Hodgkin’s disease-related mortality and reduced tolerance to chemotherapy in older patients [18]. Regarding relapse, no significant association was found between relapse and age, although some studies have shown that old age is associated with lower relapse rates [19].

Regarding stage and histological subtype, our study showed no significant association between stage, subtype, and treatment response. However, in other studies, early-stage HL was associated with better treatment response and better prognosis than advanced-stage HL [20], while histological subtypes showed identical response rates to therapy [21], which agrees with the finding in our study that there was no significant association between treatment response and histological subtype.

Dose escalation in our study was not significantly associated with gender, subtype, stage, and bulky disease; however, younger patients required dose escalation, which suggests that the ABOD regimen might be more suitable for older patients with HL. Salvage therapy is used to treat refractory/relapsed HL, according to current guidelines [22]. Despite the clinical expectation that certain baseline characteristics such as age, histological subtype, disease stage, or gender might influence the likelihood of requiring salvage therapy, our analysis did not reveal any statistically significant associations. This suggests that the need for salvage therapy in patients treated with the ABOD regimen may be driven more by individual variations in tumor biology or treatment resistance mechanisms.

Stem cell transplantation is used to treat relapse/refractory HL [23]. In our study, no significant association was found between the need for transplantation and age, gender, bulky disease, stage, or histological subtype. Generally, stem cell transplantation is recommended and feasible for elderly patients because it has a similar profile in terms of survival and toxicity in younger patients [24]. Immunotherapy agents have been developed for the treatment of HL with promising results, especially in relapsed/refractory cases. In our study, no significant association was found between the use of immunotherapy and other factors such as gender, age, or disease stage. Recent studies have shown that certain immunotherapies have superior efficacy compared with the classical ABVD regimen in patients with advanced-stage HL [14]. The limitations of this study included its retrospective design. Data collection was performed from a single center, and confounding factors that influence treatment response, such as the medical comorbidities of the patients, were included.

## Conclusions

This study evaluated the clinical outcomes of using the ABOD regimen, which includes vincristine, in the treatment of classical HL. The findings suggest that the regimen is associated with favorable treatment responses, particularly among older patients, who demonstrated higher rates of complete remission and lower need for escalation. No significant associations were found between treatment response and other variables such as gender, disease stage, or histological subtype. These results highlight the potential role of vincristine-containing regimens in specific patient subgroups and underscore the importance of individualized treatment approaches. Future prospective studies are warranted to further explore the long-term outcomes, safety profile, and optimal use of the ABOD regimen in broader patient populations.
